# El derecho al aborto legal, seguro y gratuito en Argentina: obstáculos y desafíos de la política en acto a 18 meses de su implementación (2021-2022)

**DOI:** 10.18294/sc.2023.4613

**Published:** 2023-11-24

**Authors:** Claudia C. Anzorena

**Affiliations:** 1 Doctora en Ciencias Sociales. Investigadora independiente, Consejo Nacional de Investigaciones Científicas y Técnicas, con sede en Instituto de Ciencias Humanas, Sociales y Ambientales. Coordinadora académica, Maestría en Estudios Feministas, Universidad Nacional de Cuyo, Mendoza, Argentina. canzorena@mendoza-conicet.gob.ar Universidad Nacional de Cuyo Maestría en Estudios Feministas Universidad Nacional de Cuyo Mendoza Argentina canzorena@mendoza-conicet.gob.ar; Consejo Nacional de Investigaciones Científicas y Técnicas

**Keywords:** Aborto Legal, Salud Sexual y Reproductiva, Políticas Públicas, Feminismos, Argentina, Legal Abortion, Sexual and Reproductive Health, Publics Politics, Feminisms, Argentina

## Abstract

En Argentina, en enero de 2021, entró en vigor la Ley 27610 de Interrupción Voluntaria del Embarazo, que habilita el aborto voluntario hasta la semana 14 y sin plazos en caso de violación o riesgo de la salud de la mujer, la niña o la persona gestante. Desde una perspectiva feminista y de la salud colectiva, esta Ley plantea grandes desafíos en los procesos de implementación de políticas de derechos sexuales y reproductivos en el sistema de salud. Desde una estrategia metodológica cualitativa, basada en técnicas de análisis documental, realizamos primero un análisis de los antecedentes para construir las condiciones políticas desde el activismo y lo institucional; segundo, la conquista de la ley y la formulación de su implementación; tercero, los obstáculos y barreras en el acceso institucional a la Ley; y cuarto, el desarrollo de la política en acto, haciendo hincapié en las articulaciones entre el personal de la salud y las personas funcionarias y activistas, para comprender las relaciones entre intervención estatal, prácticas y experiencias.

## Abstract (en)

In Argentina, in January 2021, Law 27610 on Voluntary Interruption of Pregnancy came into effect, allowing voluntary abortion until week 14 and without time restrictions in cases of rape or risk to the health of the woman, girl, or pregnant person. From a feminist and collective health perspective, this law poses significant challenges in the implementation processes of sexual and reproductive rights policies within the healthcare system. Employing a qualitative methodological strategy based on documentary analysis techniques, we first conduct an analysis of the background to establish the political conditions stemming from activism and institutional factors. Second, we explore the law’s acquisition and the formulation of its implementation. Third, we examine the obstacles and barriers in institutional access to the law. Fourth, we delve into the development of the policy in action, emphasizing the connections between healthcare personnel, government officials, and activists, aiming to comprehend the relationships between state intervention, practices, and experiences.

## INTRODUCCIÓN

El aborto, como evento reproductivo, implica aspectos prácticos y teóricos relevantes para la salud colectiva. En los tres ejes de análisis que propone Breilh^1^, se posiciona como un tema central para la determinación social de la salud, sobre todo en lo relativo a las relaciones desiguales entre los géneros y a su carácter histórico y cultural; para la reproducción social, sobre todo en lo relativo a las tareas de cuidado y de atención que desbordan al sistema de salud pública; y para el metabolismo sociedad-naturaleza, dado que los avances tecnológicos en sexualidad humana y reproducción permiten entender cómo las sociedades y la naturaleza interactúan y se influyen mutuamente.

En las últimas dos décadas, las prácticas políticas y experiencias de acompañamientos que el movimiento feminista fue construyendo, sumadas al éxito del uso de medicamentos para realizar abortos seguros, cimentaron las condiciones necesarias para que sea aplicable y accesible la Ley 27610 de Interrupción Voluntaria del Embarazo (Ley IVE), en el sistema de salud argentino^1^^,^^2^. Esta Ley sancionada en diciembre de 2020 y vigente desde enero de 2021, se trata de una normativa de alcance nacional que despenaliza y legaliza el aborto voluntario hasta la semana 14 de gestación y sin plazos cuando el embarazo es producto de una violación o pone en riesgo la salud de la mujer, la niña o la persona gestante. La sanción de esta ley, que marca un hito en la ampliación e integralidad de derechos, presenta grandes desafíos en los procesos de aplicación de políticas de salud sexual y reproductiva. 

Esta investigación está enmarcada en el Programa de Posdoctorado en Salud Colectiva de la Red Internacional en Salud Colectiva y Salud Intercultural (REDSACSIC). En este proyecto nos planteamos comprender los procesos de implementación de la Ley IVE entre 2021 y 2022. Específicamente, analizamos los procesos de formulación de la estrategia política, su organización en un protocolo de atención y la acción técnico-administrativa para brindar la prestación concreta. Asimismo, buscamos explorar los obstáculos, las diferencias y desigualdades en el acceso e indagar en los modos en que el Estado apela a profesionales de la salud y a las activistas, para comprender las relaciones entre intervención estatal, prácticas y experiencias. 

En este artículo compartiremos parte de los resultados en torno a las preguntas sobre ¿cómo se construyó esta política?, ¿cuáles fueron las condiciones políticas e institucionales que hicieron posible su implementación y qué características le imprimieron?, ¿cómo ingresa y se organiza la implementación en el sistema de salud?, ¿cómo recibe el personal de salud las disposiciones de la Ley IVE en su práctica cotidiana?, ¿cómo llegan las usuarias?, ¿qué rol juegan las activistas? La exposición de los resultados está organizada del siguiente modo: primero, un análisis de los antecedentes para construir las condiciones políticas desde el activismo y lo institucional; segundo, la conquista de la ley y la formulación de su implementación; tercero, los obstáculos y barreras en el acceso de la Ley; y cuarto cómo se desarrolla la política en acto entre resistencias ideológicas, barreras concretas y presiones de activistas. Analizar las dimensiones histórica e institucional de la implementación de la Ley IVE es un aporte a la construcción de conocimiento sobre cómo ampliar derechos y que su garantía sea asequible.

## METODOLOGÍA

El diseño epistemológico parte de una perspectiva situada, feminista y articulada con la salud colectiva para el análisis de las políticas de derecho a la salud sexual y reproductiva. La estrategia metodológica es de tipo cualitativa. La técnica de recolección de datos, basada en el análisis documental, permitió construir un corpus empírico para analizar los procesos de formulación de estrategias políticas, la organización de la atención y la acción técnico-administrativa de la política en acto, y explorar los obstáculos y desigualdades en el acceso a la ley. Mi participación como feminista en Mendoza desde 2000 y como activista, desde 2005, de la Campaña Nacional por el Derecho al Aborto Legal, Seguro y Gratuito me otorga una posición privilegiada y una incursión prolongada en el campo. 

En torno a la planificación e implementación de políticas públicas se produce un importante repertorio de material documental: proyectos de ley, guías de procedimientos, protocolos de actuación, manuales operativos e informes de gestión y monitoreos. La recolección consistió en el acopio de textos relativos a la Ley IVE para conocer la forma de organización de la implementación. A esta compilación se suman los informes de activistas de diferentes organizaciones regionales y redes, presentados en la última reunión plenaria de la Campaña Nacional por el Derecho al Aborto Legal, Seguro y Gratuito de diciembre 2022, y monitoreos sociales realizados por las organizaciones de la sociedad civil Católicas por el Derecho a Decidir (CDD-Córdoba), Mujeres X Mujeres, el Proyecto Mirar del Centro de Estudios de Estado y Sociedad (CEDES) y el Grupo FUSA para la Atención de Adolescentes y Jóvenes. Si bien este corpus está nutriéndose permanentemente, logra dar un panorama de lo que ocurre en los diferentes territorios con la implementación de la ley (Tabla 1). 

**Tabla 1:** Cuerpo documental recopilado sobre interrupción voluntaria del embarazo. Argentina, 2021-2023.

Tipo de fuente documental	Autoría	Título	Organismo responsable	Año	Acceso
Documentos oficiales	Dirección Nacional de Salud Sexual y Reproductiva	Informe de gestión anual 2022	Dirección Nacional de Salud Sexual y Reproductiva, Secretaría de Acceso a la Salud, Ministerio de Salud de la Nación	2023	Acceso al documento
	Oficina de presupuesto del Congreso de la Nación	Caracterización del sistema de salud argentino	Oficina de presupuesto del Congreso de la Nación	2021	Acceso al documento
	Valeria Isla (coord.), Sonia Ariza Navarrete, Eunice León, Guillermo Ortiz, Mariana Romero, Raffaela Schiavon.	Protocolo para la Atención Integral de las Personas con Derecho a la Interrupción Voluntaria y Legal del Embarazo IVE-ILE: actualización 2022	Ministerio de Salud de la Nación, Dirección Nacional de Salud Sexual y Reproductiva	2022	Acceso al documento
	República Argentina	Ley 27610 Acceso a la Interrupción Voluntaria del Embarazo (IVE)	Honorable Senado y Cámara de Diputados de la Nación Argentina	2020	Acceso al documento
Informes del Centro de Estudios de Estado y Sociedad	Mariana Romero, Silvina Ramos, Agustina Ramón Michel, Briana Keefe-Oates, Emilia Rizzalli	Proyecto mirar: a un año de la ley de aborto en Argentina	CEDES, Idis Reproductive Health, Proyecto Mirar	2021	Acceso al documento
	Silvina Ramos, Edgardo Ábalos, Caitlin Gerdts, Brianna Keefe-Oates, Mercedes Krause, Agustina Ramón Michel, Mariana Romero	Calidad: el desafío a dos años de la ley de aborto	CEDES, Idis Reproductive Health, Proyecto Mirar	Acceso al documento
	Mariana Romero, Agustina Ramón Michel, Mercedes Krause, Briana Keefe-Oates, Edgardo Ábalos, Silvina Molina, Silvina Ramos	Reporte anual 2022: Los rumbos de la experiencia argentina con el aborto legal	CEDES, Idis Reproductive Health, Proyecto Mirar	2023	Acceso al documento
Monitoreos de organizaciones de la sociedad civil	María Alicia Gutiérrez, Josefina Sabaté, Rosa Mahffuz, (coord.).	1º Monitoreo social de Centros de Atención Primaria y Secundaria de la Salud: Acceso y calidad de atención de la salud sexual y reproductiva e Interrupción legal y voluntaria del embarazo en Chivilcoy – Prov. De Buenos Aires	Campaña Nacional por el Aborto Legal, Seguro y Gratuito Chivilcoy, FUSA	2022	Acceso al documento
	María Alicia Gutiérrez, Rosa Mahffuz, Josefina Sabaté, (coord.).	1º Informe Monitoreo Social Ampliando el ejercicio de derechos General Belgrano, Prov. de Buenos Aires	FUEGAS feminismo y diversidad, FUSA	2022	Acceso al documento
	María Alicia Gutiérrez, Josefina Sabaté, Rosa Mahffuz, (coord.).	Monitoreo social de servicios de salud de Monte	Vivas Colectiva Feminista, FUSA	2022	Acceso al documento
	María Alicia Gutiérrez, Josefina Sabaté y Baudron, Rosa Mahffuz, (coord.).	2° Informe de monitoreo social en servicios de salud públicos: Acceso y calidad de la atención de la salud sexual y reproductiva e interrupción voluntaria y legal del embarazo en Morón	Conurbanas Colectiva Transfeminista, FUSA	2022	Acceso al documento
	Colectiva Feminista Suipacha, FUSA	1º Monitoreo sobre Políticas Sanitarias y Salud Sexual en el Municipio de Suipacha Prov. Bs. As.	Colectiva Feminista Suipacha, FUSA	2022	Acceso al documento
	María Alicia Gutiérrez, Josefina Sabaté, Rosa Mahffuz (coord.).	1º Monitoreo social: Ampliando el Ejercicio de Derechos	FUSA, Consejo Municipal de las Mujeres de Tornquist	2022	Acceso al documento
	Católicas por el Derecho A Decidir Argentina, Mujeres X Mujeres	Monitoreo social sobre anticoncepción y aborto en Santiago del Estero	Católicas por el Derecho a Decidir Argentina, Mujeres X Mujeres	-	Acceso al documento
Monitoreos de organizaciones de la sociedad civil	Católicas por el Derecho A Decidir Argentina, Mujeres X Mujeres	Monitoreo social sobre anticoncepción y aborto en Tucumán	Católicas por el Derecho a Decidir Argentina, Mujeres X Mujeres	-	Acceso al documento
	Soledad Deza, Lila Aizenberg	Monitoreo social sobre anticoncepción y aborto en Jujuy	Mujeres X Mujeres	2022	Acceso al documento
	Católicas por el Derecho a Decidir Argentina	Acceso a la salud sexual y reproductiva en la provincia de La Rioja	Rodando Derechos: Fortalecimiento de los Equipos de Salud de las Provincias Argentinas, Católicas por el Derecho a Decidir Argentina, Red de Organizaciones de La Rioja	2022	Acceso al documento
Informes de Regionales y Redes para la reunión plenaria del 3 y 4 de diciembre de 2022	Regionales: San Luis, Villa Mercedes, Merlo y Traslasierras (Provincia de San Luis); Rio Negro y Bariloche (Río Negro); Chubut, Córdoba; Foro Pampeano por los DDSSyRR (La Pampa); Rosario (Santa Fe); Salta; La Plata, Regional Zona Sur GBA, Conurbano Norte, Marcos Paz (Provincia de Buenos Aires). RUDA (Red de Cátedras en Universidades Públicas Nacionales sobre ESI y Derecho al Aborto) y Comisión Federal Libres Las Queremos	Archivos de circulación interna para la reunión plenaria federal del 3 y 4 de diciembre de 2022	Campaña Nacional por el Derecho al Aborto Legal, Seguro y Gratuito	2022	No publicado

Fuente: Elaboración propia.

Para analizar los datos, marcamos las tres etapas de la planificación social que propone Caroline Moser^3^:

- Formulación de la política (qué hacer): es el proceso de toma de decisiones acerca de cómo asignar recursos para atender las necesidades de la sociedad, que concluye en la formulación de una estrategia de política.
- Planificación (cómo hacerlo): es el proceso de organización de la implementación de la política que a menudo concluye en un plan.
- Puesta en práctica de la implementación (qué se hace): es el proceso de acción administrativa para entregar el programa diseñado, que a menudo resulta en un producto o servicio acabado.

Este esquema permite analizar la implementación como un proceso de toma de decisiones en curso, determinado por el contenido del programa que se sigue y por la interacción entre los diferentes actores implicados en un contexto político-administrativo dado. La distinción entre diferentes etapas es clave para precisar el tipo de problemas e identificar el grado en que sus trabas son políticas o técnicas, y actuar en consecuencia^3^. 

Las feministas, a través de sus prácticas de formación y acompañamiento de abortos seguros y autogestivos, crearon las condiciones para que la implementación de la Ley IVE en el sistema de salud sea posible, lo que tiene un impacto en su efectividad y alcance a pesar del poco tiempo de su reglamentación. Este *background* diferencia la Ley IVE de la trayectoria de otras leyes que han sido muy accidentadas en su implementación porque, a pesar de los obstáculos y desigualdades territoriales y de cobertura, las mujeres y otras personas con capacidad de gestar están accediendo a la práctica. Entonces este análisis nos dará claves para potenciar esta y otras leyes de ampliación de derechos, a la vez que sentará bases para establecer las articulaciones y las interconexiones entre las distintas modalidades en que las políticas de salud dan forma, modelan y/o regulan las experiencias de interrupciones de embarazo de mujeres y personas con capacidad de gestar. 

## RESULTADOS

### Antecedentes activistas e institucionales: construir las condiciones políticas

En 2005, feministas de diferentes puntos de país se organizaron para impulsar la despenalización y legalización del aborto voluntario en Argentina, bajo el paraguas de la Campaña Nacional por el Derecho al Aborto Legal, Seguro y Gratuito (la Campaña). Esta se lanzó el 28 de mayo de 2005 en diferentes puntos del país, con el objetivo de conseguir la sanción de una ley que permitiera la interrupción voluntaria del embarazo. A la vez emprendió un intenso proceso de instalación social de la problemática como un asunto de salud pública, justicia social y derechos humanos, y exigió el cumplimiento de los abortos permitidos según el Código Penal^4^^)^ de Argentina. La Campaña acordó el lema “educación sexual para decidir, anticonceptivos para no abortar, aborto legal para no morir”, que hacía referencia al contexto de surgimiento. 

En 1995, en la IV Conferencia Mundial de la Mujer de Beijing se reconoció a la salud sexual y reproductiva como parte de los derechos humanos y la igualdad de género. Si bien algunas provincias avanzaron en la creación de programas para atender la salud reproductiva, no fue hasta 2003 que se sancionó la Ley 25673 que crea el Programa Nacional de Salud Sexual y Procreación Responsable para “garantizar a toda la población el acceso a la información, orientación, métodos y prestaciones de servicios referidos a la salud sexual y procreación responsable”. En esos años se dieron debates en torno a la responsabilidad del sistema educativo para dar a conocer y garantizar los derechos de las niñas, los niños y adolescentes, y en 2006 se sancionó la Ley 26150 que establece el derecho a recibir Educación Sexual Integral (ESI) y crea el Programa Nacional de ESI, con el propósito de garantizar este derecho en todas las escuelas del país. 

En marzo de 2012, la Corte Suprema de Justicia de Argentina dio a conocer el fallo histórico “F., A. L. s/ medida autosatisfactiva”^5^, para aclarar la interpretación de la causal violación del Código Penal de la Nación Argentina, basada en la Constitución Nacional y en los tratados internacionales a los que Argentina suscribe. El documento señala que el aborto no es punible en caso de violación, independientemente de las facultades mentales de la víctima. Además, trasciende la sola aclaración e indica que no garantizar la interrupción de un embarazo producto de una violación a quien lo solicita es discriminatorio y violatorio de los derechos humanos, y exhorta a los gobiernos provinciales a implementar y garantizar la aplicación de protocolos hospitalarios para la atención de los abortos en las causales que son legales, sin dilación ni judicialización, con la sola declaración jurada de las mujeres ante el médico. También indica la existencia y plena vigencia de la *Guía de atención de abortos no punibles* del Ministerio de Salud de la Nación. Este camino comenzó en 2007, cuando el Ministerio de Salud elaboró esta guía como respuesta a las obstaculizaciones de una serie de solicitudes de abortos enmarcados en las causales de no punibilidad, que la Campaña, en diferentes puntos de pías, venía acompañando y exigiendo su cumplimiento^4^^,^^6^^,^^7^. 

En esta doble incidencia, que tejía redes tanto en lo social como en lo institucional y legal, había una gran preocupación por lo que ocurría en la ilegalidad y sus consecuencias con las mujeres y otras personas con capacidad de gestar. Además de las acciones de cabildeo en el Congreso y de atracción de movimientos sociales, sindicatos y todo actor social sensibilizado por la temática, se profundizaron las acciones de acompañamiento a quienes necesitaban abortar. Las feministas venían compartiendo datos de médicos y médicas amigables y accesibles en diferentes territorios, cuando se conoció la existencia del misoprostol, una droga que provoca abortos. Entonces empezaron a estudiar su uso: cómo funcionaba, su seguridad y cómo se podía acceder. Las Feministas Inconvenientes, la Campaña y otras colectivas comenzaron a elaborar folletos y a repartirlos en diferentes actividades, encuentros y movilizaciones. En 2009, Lesbianas y Feministas por la Descriminalización del Aborto activaron una línea telefónica para brindar información sobre el uso seguro de misoprostol y en 2010 publicaron un manual que tuvo gran difusión^8^^,^^9^. La Línea no alcanzaba a responder la gran demanda de información y era un número de larga distancia para quienes no residían en la CABA. Esta situación llevó a que las personas que necesitaban asesoramiento se comunicaran con activistas locales y que las colectivas de las provincias se organizaran para crear sus propios canales de comunicación, a través de los cuales daban información de cómo usar las pastillas, qué era esperable sentir y las señales de alarma para reconocer las complicaciones. Dar información rápidamente atrajo a una gran demanda de acompañamientos durante el proceso, entonces se organizaron para hacer guardias telefónicas mientras las mujeres estaban abortando, por si surgían dudas o inquietudes o para saber cómo actuar en una guardia médica sin incriminarse. Ante el incremento de llamadas, organizaron consejerías grupales que, en algún lugar público como plazas o cafés, reunían a las interesadas y a dos o tres activistas para brindar un acompañamiento cuidado que consistía en información, contención y creación de mecanismos para acceder a las pastillas en los lugares donde se iba restringiendo o encareciendo la oferta. 

Esta forma de acompañar, emprendida por un gran número de colectivas que formaban parte de la Campaña en sus provincias, dio lugar en 2012 a la creación de la Red de Socorristas y a su multiplicación en gran parte del país. Además, y con las habilitaciones que implicó el fallo FAL^5^, las activistas articulaban con profesionales de la salud amigables, para derivar a personas dentro de las causales de ILE, para consultas sobre situaciones contraindicadas con el uso del misoprostol, y para las consultas pre o postaborto. Incluso, las socorristas recibían mujeres derivadas por médicas y médicos, que evitaban realizar los abortos. A pesar de que las articulaciones entre activismo y sistema de salud eran tensas y difíciles, las activistas se sirvieron de lo que este les habilitaba para que las mujeres se realizaran los abortos de manera segura y cuidada, mientras el sistema de salud hacía la vista gorda y se desligaba del problema. 

En 2018, se desarrolló el primer debate parlamentario del Proyecto de Ley de Interrupción Voluntaria del Embarazo elaborado por la Campaña. La gran movilización de la Marea Verde y las decenas de intelectuales, activistas, figuras públicas y sanitaristas que transitaron por el debate público, aunque no logró la sanción en ese entonces, conmovió todas las esferas sociales e introdujo en agenda la problemática. Al calor de este debate, diferentes profesionales de la salud -muchas que integraban la Campaña- se aglutinaron en la Red de Profesionales por el Derecho a Decidir y comenzaron a activar dentro del sistema para ampliar las posibilidades de garantizar los abortos en las causales que establecía el Código Penal, con una definición de salud amplia. Ese año, la Administración Nacional de Medicamentos, Alimentos y Tecnología (ANMAT) aprobó el uso obstétrico del misoprostol y se incrementaron los abortos legales realizados en los servicios de salud pública, atendiendo a procedimientos delineados por el Ministerio de Salud de la Nación que se condecían con lo indicado por organismos internacionales, con lo que las activistas venían a su vez construyendo con sus saberes y prácticas. 

Si como señala Paim “las formas en que las sociedades identifican sus problemas de salud, buscan su explicación y se organizan para enfrentarlos varían históricamente y dependen de factores determinantes estructurales económicos, políticos e ideológicos”^10^, estas prácticas de construcción de saberes y de articulación con profesionales de la salud contribuyeron a crear las bases para que, una vez legalizada la IVE, el sistema de salud tuviera mecanismos ya en funcionamiento para garantizar el acceso a una práctica históricamente negada. Las feministas se organizaron para crear las condiciones para que el aborto seguro sea accesible aún en la clandestinidad, pero entendían que se necesitaba la legalidad y la gratuidad, cada una condición necesaria pero no suficiente. La legalidad permite la inclusión como derecho que deben garantizar los servicios de salud. El sistema de salud tiene las capacidades para garantizar la gratuidad para las usuarias, y la gratuidad universaliza el servicio para todas las personas independientemente de la cobertura en salud o de los recursos económicos.

La gratuidad es una condición posible en Argentina debido al funcionamiento del sistema de salud. Si bien tiene importantes deficiencias porque es altamente desigual y está fragmentado entre subsistemas, tipos de cobertura y territorios provinciales, el subsistema público tiene capacidad para absorber la demanda de atención de quienes no tienen cobertura o que viven en localidades rurales o alejadas de los centros urbanos, a través de su red de hospitales y de Centros de Atención Primaria de la Salud o CAPS^11^^,^^12^^,^^13^. 

En 2019, durante la campaña electoral, el candidato Alberto Fernández manifestó como compromiso avanzar con la legalización del derecho al aborto, ya que entendía que era una demanda justa. A inicios de 2020, ya con Fernández como presidente, se presentó un proyecto de ley elaborado por letradas y funcionarias del gobierno que tomaba en gran parte el trabajo de la Campaña. El segundo debate se dio en el marco restrictivo de las medidas sanitarias para enfrentar la pandemia por covid-19, pero no impidió que en diciembre de 2020 se movilizaran cientos de miles para apoyar la sanción de la ley.

### La conquista de la ley: la formulación e institucionalización de la política

En diciembre de 2020 se sancionó la Ley 27610, puesta en vigor en enero de 2021 en todo el territorio nacional. Se trata de una ley de orden público y, por ende, de aplicación obligatoria en todo el país, sin necesidad de que las provincias adhieran. Esta ley establece un sistema mixto de plazos y causales. Hasta la semana 14 de gestación inclusive se puede solicitar el aborto sin dar motivos, con el solo consentimiento informado y sin plazo para las causales de salud y violación. 

Para la organización de la implementación, desde el Programa Nacional de Salud Sexual se han elaborado una serie de guías técnicas o protocolos de acción. Estas guías no son documentos meramente técnicos descriptivos, sino que tienen un desarrollo histórico que refleja el cambio en las formas de entender las normas y de definir el derecho al aborto como una necesidad legítima de la ciudadanía, pasando de abortos no punibles a interrupción legal del embarazo (ILE). En 2007, se publica la primera Guía que hace referencia a “abortos no punibles”, como se denominaban los abortos permitidos en el Código Penal. Esta Guía hace hincapié en la no judicialización y el no requerimiento de denuncia policial por causal salud y violación. Está basada en las directrices de reducción de daños en marcos legales restrictivos de la Organización Panamericana de la Salud, y en la atención de los abortos en curso y el postaborto. En 2010, se actualiza y reedita en lo relativo a la información médica, bioética y legal. En 2012, el fallo FAL^5^ establece que lo que no está prohibido está permitido y que se debe garantizar. A la luz de este fallo, en 2015, se actualiza la Guía y se le modifica el nombre a “Protocolo para la atención integral de las personas con derecho a la interrupción legal del embarazo (ILE)”^14^, enmarcando en la legalidad las causales de salud y violación. En este protocolo se incluye el aborto medicamentoso con misoprostol solo y con mifespristona y misoprostoles combinados^14^. En septiembre de 2016, se introduce una nota aclaratoria a partir de la aprobación del nuevo Código Civil y Comercial, que amplía la edad requerida para el ejercicio autónomo de los derechos sexuales y reproductivos de niñas, niños y adolescentes y las condiciones para el ejercicio autónomo de derechos para las personas con discapacidad. En 2018, el entonces ministro de salud Adolfo Rubinstein defendió activamente la aprobación de la ley. En 2019, el protocolo es editado dos veces, la primera vez por la gestión Rubinstein y la segunda con la nueva gestión de González García, que lo reforzó firmando en nombre del Ministerio de Salud, la Resolución 1/2019^15^ para darle mayor legitimidad.

En 2020, por Decisión Administrativa 457/2020^16^, se crea la Dirección Nacional de Salud Sexual y Reproductiva (DNSS) con el objetivo de promover la salud sexual y reproductiva de la población. Los objetivos estratégicos son garantizar el acceso a métodos anticonceptivos, a la interrupción voluntaria del embarazo e interrupción legal del embarazo, de acuerdo con la Ley 27610, a la prevención y detección de abusos sexual y embarazos forzados, y a la promoción de derechos sexuales y reproductivos de personas con discapacidad. La DNSS trabaja a partir del Programa Nacional de Salud Sexual y Reproductiva (PNSSR) y el de Prevención del Embarazo No Intencional en la Adolescencia (Programa ENIA). La gestión de estos programas se encuentra organizada territorialmente por provincias y con líneas transversales de trabajo en articulación con los programas de salud sexual y reproductiva en cada provincia. Entre sus acciones está la provisión de insumos necesarios en los establecimientos de salud de todo el país, la producción de material educativo y protocolos de actuación, la formación y actualización de equipos de salud, y la difusión de información para toda la población a través de diversos materiales de comunicación y promoción de la línea 0800 Salud Sexual, que es un número gratuito al que se puede llamar para pedir información o para reclamar la falta de atención^17^.

En 2021, fue presentado el “Protocolo para la atención integral de las personas con derecho a la interrupción voluntaria y legal del embarazo”^18^ para el cumplimiento de la Ley 27610, que fue actualizado en 2022 cuando la ANMAT incorporó la autorización del uso y producción de mifepristona. El protocolo señala que su objetivo principal es “ofrecer una guía a los equipos y establecimientos de salud” y que “está diseñado en base a la comprensión fundamental de que todo el personal de salud (incluyendo el administrativo y de seguridad) es responsable de garantizar y no obstruir el derecho a interrumpir un embarazo”^18^. Consta de dos partes: la primera es el marco legal, en el que se desarrolla la normativa nacional e internacional y, la segunda, son los mecanismos de atención de las mujeres y otras personas gestantes que solicitan la práctica. El protocolo explica detalladamente los principios rectores de la normativa para el ejercicio del derecho a la IVE/ILE y la elaboración del consentimiento informado para personas adultas y con plenas facultades, para niñas y adolescentes de acuerdo con la edad, para personas con discapacidad, personas con sentencia judicial vigente de restricción de la capacidad y personas en situación de imposibilidad absoluta de expresar su voluntad. En todos los casos prima la voluntad de la titular del derecho. 

Se establecen los plazos para realizar la práctica y las responsabilidades institucionales y profesionales y de todo el personal que trabaja en un servicio de salud, así como las pautas que delimitan el ejercicio de la objeción de conciencia. Solo pueden objetar los profesionales que tengan implicación directa en el procedimiento y no quienes tienen participación indirecta como ecografistas, personal de laboratorio o administrativo, de seguridad o maestranza. La objeción no puede ser institucional y deben brindarse los recursos para derivación de buena fe y sin obstaculización. Se debe ejercer tanto en el ámbito público como privado, y no se puede argüir en caso de urgencia o de consulta posaborto, es decir, solamente pueden negarse por creencias personales y declaradas, y deben ejercerla en todos los ámbitos donde practiquen la medicina. Las obras sociales y la medicina privada pueden elaborar sus propios protocolos, pero cumpliendo la ley.

En cuanto al marco legal, es interesante observar que no solo da pautas para los efectores y agentes de salud sobre sus obligaciones, sino que también brinda tranquilidad a las personas que sí se disponen a realizar la práctica y cuáles son los parámetros de la legalidad que las protegen. Además, limita los abusos de profesionales que no quieran asumir la responsabilidad por desidia o desinterés de involucrarse con una práctica que produce discrepancias, o que buscan llevar el beneficio económico al sector privado.

En cuanto a los procesos de atención, el protocolo establece que se atiende por demanda espontánea, es decir de las mujeres y personas gestantes que solicitan la intervención en el marco de la ley. Todo el personal de salud debe cumplir con las pautas de trato digno y brindar atención de calidad dentro de los diez días de la solicitud. Desde ordenanzas, personal administrativo, ecografistas, laboratorios, hasta enfermeras, anestesistas, obstetras, psicólogas, trabajadoras sociales y personal médico. Se les debe brindar información adecuada, veraz y respetuosa de la autonomía y las decisiones en las condiciones que establecen a este tenor la Ley 26485 de protección integral para prevenir, sancionar y erradicar la violencia de género y contra las mujeres de género, y la Ley 26529 que establece los derechos de los y las pacientes en su relación con los profesionales e instituciones de la salud, que rige el derecho a la autonomía de la voluntad, la información y la documentación clínica.

El proceso se inicia con una consejería donde se brinda la información y se realizan anamnesis, evaluación física y estudios complementarios solo si es necesario. Todo se debe registrar en la historia clínica y se adjunta el consentimiento informado. En caso de causal salud o violación se adjuntan las interconsultas y la declaración jurada de lo ocurrido; en ningún caso se necesita orden judicial, ni siquiera para la preservación de la prueba. La o el profesional debe evaluar con la persona el método que considere más adecuado, de acuerdo con la edad gestacional, las preferencias de la interesada y las posibilidades del centro de asistencia. La edad gestacional se puede establecer a partir de la fecha de la última menstruación y del examen pélvico bimanual y abdominal, y el reconocimiento de los signos del embarazo. Los análisis de laboratorio y las ecografías no son requerimientos: se pueden solicitar complementariamente, pero no pueden ser un impedimento para la intervención.

En general se trata de un procedimiento de baja complejidad posible de resolver en el sistema de atención primaria y de manera ambulatoria, sobre todo en embarazos de menos de catorce semanas. El método medicamentoso puede ser con misoprostol solo o combinado con mifepristona. El método quirúrgico puede ser aspiración manual endouterina (AMEU) y dilatación y evacuación. Nunca se recomienda el legrado por riesgoso y obsoleto. La guía explica detalladamente cómo, cuándo, dónde y por quién llevar a cabo cada uno de estos procedimientos. En el caso de los embarazos de menos de doce semanas, en general se elige el procedimiento medicamentoso: se les entregan a las mujeres los insumos y se les dan las indicaciones de uso y los síntomas de alarma. En todos los casos se les debe proponer una consulta posterior y sugerir el uso de algún método anticonceptivo. Todo lo requerido previo, durante y posterior al procedimiento está contemplado en la gratuidad del servicio^18^.

El Protocolo es una guía de actuación detallada en sus diferentes niveles: legales, de servicios de atención de calidad, e incluso de procedimientos específicos y actualizados. Este detalle y las diferentes reediciones muestran un trabajo de años de estudio, de experiencia y de conocimiento del terreno, tanto de las determinaciones sociales de la problemática como del funcionamiento del sistema de salud. Sin embargo, en los procesos organizacionales, cuando ingresan en el accionar las prácticas concretas y las relaciones de fuerza de los sujetos y de las instituciones, los efectos de la implementación se vuelven múltiples y poco controlables^19^^,^^20^. A continuación, abordamos algunos de los obstáculos que se presentan para la implementación de la ley y para el acceso efectivo de las mujeres y personas gestantes.

### Obstáculos y barreras en el acceso a la Ley

En los procesos de implementación de la Ley IVE observamos tres tipos de obstáculos: los que son propios del sistema de salud pública y estructurales de todo el funcionamiento del Estado; los que son propios de la práctica: acceso, información, formación de los profesionales en la normativa y en los procedimientos; y obstáculos subjetivos: temores, falta de información, resistencias, miedo a “que se enteren”.

Rodríguez Gustá^21^ señala que los problemas y obstáculos en el desarrollo de las políticas con perspectivas de género tienen que ver no solo con las resistencias de las autoridades para reconocer los derechos de las mujeres y colectivos LGTTBIQ+ como aspectos primordiales y urgentes en la intervención estatal, sino también con los problemas en las estructuras y las capacidades estatales para dar respuesta a los problemas concretos de la población. Estos problemas se reflejan en la falta de recursos adecuados, la inercia burocrática, las resistencias y la interpretación de las relaciones desiguales de género como un proceso ajeno a las propias prácticas institucionales y profesionales.

El proceso al que da lugar la Ley 27610 plantea el desafío de articular con un sistema de salud que trae sus deficiencias y arrastra desigualdades históricas. Estas determinaciones son tanto estructurales y culturales como distributivas, en un sistema federal donde las provincias tienen las responsabilidades de gestionar la salud sin los recursos adecuados, producto de la descentralización de la década de 1990. A esto se suman otros determinantes como las resistencias del personal de salud y de las instituciones ante prácticas que implican cambios en su entorno y su actuar.

El sistema de salud argentino es un sistema segmentado, con desigualdades territoriales y geográficas, con límites para el acceso por la deficiencia del sistema de transporte y las condiciones de empleo. La hiperespecificación de las especialidades médicas, la precarización laboral del personal de salud en relación de dependencia, y las deficiencias en la infraestructura y en la distribución de insumos impactan en las posibilidades y en la calidad de atención de los servicios^11^.

La diferenciación de los subsistemas público, seguridad social obligatoria u obra social y privado también tiene impacto en la aplicación de la ley. Pero contrariamente a lo que ocurre con otros procedimientos, el subsistema público parece el más accesible, en cuanto a que circula mayor información sobre qué centros realizan el procedimiento y qué requisitos solicitan. Además, dan información a la DNSS, lo que les permite optimizar la distribución de insumos y brindar formaciones y actualizaciones a demanda de las provincias. En el caso de las obras sociales y la medicina privada, aplican sus propios protocolos si lo hacen y no informan al MINSAL sobre sus prácticas, lo que hace difícil realizar monitoreos y llevar estadísticas adecuadas^22^.

El sistema de salud tiene tres ejes de actuación transversales a todas las jurisdicciones: de promoción, de prevención, y de atención de la salud. La mayor parte de los recursos de esta red se aplican para las acciones de atención de la salud, lo que se replica en las acciones de la DNSS. Sin embargo, en el informe de la Oficina de Presupuesto del Congreso^11^ advierten una marcada variabilidad entre las provincias en cuanto a la disponibilidad de establecimientos y profesionales de la salud en relación con sus poblaciones. Por ejemplo, hay una importante brecha entre la jurisdicción con mayor cantidad de médicos y médicas cada 100.000 habitantes y la de menor cantidad, la cual es de siete veces. En cuanto a la cantidad de establecimientos de salud, la jurisdicción con mayor cantidad posee 4,7 veces más que la que menos tiene, destacándose en estas últimas una amplia presencia estatal ya que la oferta privada está focalizada en las áreas más populosas. Si bien los tres subsistemas abarcan a toda la población del país, esto no implica que la oferta sea equitativa e igualitaria. En la Conferencia Regional de CLACAI de junio 2023, el CEDES destacó que las desigualdades estructurales del sistema de salud argentino y “el aseguramiento de insumos, la rendición de cuentas y el foco en los cambios de regulaciones subnacionales y de orden administrativo […] imponen obstáculos a la plena implementación de la Ley 27610”^23^.

Si articulamos el informe de la Oficina de Presupuesto del Congreso^11^ con el informe de gestión de la DNSS^18^, observamos que las provincias con mayor proporción de establecimientos que ofrecen salud privada son las que desde el sistema público han informado más abortos, además de ser las más populosas: provincia de Buenos Aires, Ciudad Autónoma de Buenos Aires, Tucumán, Santa Fe, Córdoba y Mendoza. Y que las provincias con mayor proporción de establecimientos de salud públicos son las que menor cantidad de abortos informan a la DNSS: Catamarca, La Rioja, Santiago del Estero, Formosa y Misiones. Con la excepción de Jujuy y Salta que informan mayor cantidad de abortos y tienen un sistema público con gran presencia^11^^,^^18^. 

La DNSS, en coordinación con los responsables de salud sexual provinciales, han trabajado en la ampliación de la capacidad para resolver problemas del sistema de salud y en la remoción de las barreras de acceso. En este sentido, se han ampliado las capacitaciones para el personal de salud y se han reforzado los materiales de difusión e información. En el Informes del CEDES^22^ se señala que entre 2020 y 2022 se han casi duplicado la cantidad de servicios informados del subsector público que atienden IVE/ILE. Sin embargo, en los informes de las activistas y en los monitoreos sociales se señala que la folletería y cartelería es muy irregular en los servicios y que en muchos no hay información exhibida sobre aborto, y sí la hay sobre enfermedades y cuidado del embarazo.

Desde la DNSS se ha fortalecido la compra y provisión de insumos, y se ha realizado distribución de equipamiento para hacer AMEU y además protocolos combinados^17^^,^^22^. Del protocolo combinado, la cantidad es limitada entonces se usa en casos específicos, por ejemplo, en algunas provincias se entrega cuando no funcionó el protocolo solo con misoprostol. El protocolo combinado tiene varias ventajas, es más sencillo de administrar, tiene efectividad y produce menor cantidad de síntomas.

Por su parte la ANMAT autorizó la producción nacional de mifepristona, y se mantiene la producción pública del medicamento misoprostol. Han trabajado en la actualización del Protocolo y en los informes de gestión periódicos. El CEDES^22^ señala que el número de abortos realizados en el subsector público se incrementó en un tercio entre 2021 y 2022 en el sistema público, que es el único que informa (de 73.487 en 2021 a 96.664 a 2022).

Lo que observamos, es que, en dos años, a pesar de los obstáculos estructurales, desde la DNSS y las provincias, han llevado a cabo un importante trabajo para ampliar el acceso, lo que se ve reflejado en el 30% de crecimiento de la cantidad de abortos informados y se plantean como desafío mejorar la calidad de la atención. Si bien esta cifra tiene falencias, el incremento es esperable hasta alcanzar un estancamiento cercado a las estimaciones de alrededor de 500.000 abortos anuales en la clandestinidad.

### La política en acto: resistencias y articulaciones entre el personal de salud, funcionarias, funcionarios y activistas

El sistema de salud funciona en torno a normas y objetivos preestablecidos, pero también a encuadres ideológicos que configuran las prácticas de quienes allí trabajan. Estos encuadres son propios del espacio o de la formación profesional, pero también son aportados por los *habitus* de los sujetos que interactúan. Se convierte así en un campo de relaciones de fuerza y de lucha por ganar capital social o imponer el propio^24^.

Los espacios institucionales de salud sexual encargados de la implementación de la ley están mayormente encuadrados en los ministerios de salud provinciales. A su vez, muchos de estos están en las áreas de maternidad e infancias con profesionales formados para las tareas vinculadas con el cuidado del embarazo y de las niñeces y no con la garantía de los derechos sexuales y reproductivos. La interrupción del embarazo, e incluso el uso de métodos anticonceptivos, implican resistencia a un mandato que algunas personas se sienten llamadas a preservar, y entonces entra en colisión con los derechos de las usuarias. En esta línea hay servicios o instituciones donde se aísla o se maltrata a quienes realizan abortos, por ser una práctica estigmatizada en el campo de la obstetricia. Es así como quienes no tienen una convicción que les permita enfrentar las arremetidas ideológicas prefieren “no meterse en problemas” y no tener que lidiar con ataques.

La resistencia se traduce también en negarse a tomar las formaciones para actualizarse en las nuevas normas y prácticas. Otros que, pudiendo, eligen no ejercer la objeción de conciencia como se indica, para tener formas más efectivas de negar el derecho a las usuarias. Para el personal indirecto, un modo de desafiar el cumplimiento es maltratar, desinformar o retacear los insumos e incluso los turnos. 

Permitir el aborto voluntario y exigir a todo el personal de salud que lo ofrezca dentro de sus prácticas profesionales, técnicas o administrativas implica introducir un cambio en ese campo y realizar una práctica con una carga ideológica que se confunde con el orden de las creencias personales. Es decir, realizar abortos significa una transformación tanto en el ejercicio como en las concepciones sobre esas prácticas. No se trata de un procedimiento que se introduce “neutralmente” como puede ser un nuevo tratamiento o algunas actividades preventivas, sino de modificar el trasfondo ideológico de personas que quizá no tenían interés en involucrarse en una práctica que fue sistemáticamente expulsada y/o marginada del sistema: expulsada en cuanto decían “no hacemos eso” o “es ilegal” y las mujeres se las rebuscaban por sus medios, o marginada porque las excepciones tenían que ser justificadas médicamente y eran las y los profesionales quienes establecían cuándo cabía la causal. Es notable cómo cambia la aceptación de una práctica cuando pasa de ser una decisión médica a ser voluntad de las mujeres^25^. 

Las barreras geográficas hacen referencia a las extensiones de los territorios provinciales, que implican grandes distancias para llegar a los centros de salud y, a veces, por caminos poco transitables por su estado, por los accidentes geográficos o por condiciones climáticas adversas para moverse. Esta problemática, que se extiende a la atención primaria, no solo es una barrera para las usuarias, sino también para la organización de la implementación. Ocurre que parte del personal de salud de zonas rurales, aisladas o inhóspitas reside a largas distancias de los servicios, y muchas veces los medios de transporte públicos no llegan o simplemente el salario y las condiciones no compensan el esfuerzo. Señalan que deben realizar tareas que no les corresponde o asumir lo que otras personas se niegan a realizar. Es así como el ausentismo, la rotación de personal, la variabilidad de experticia del personal contratado, la pluriactividad y la sobrecarga de tareas impactan negativamente en la atención. Las dificultades en la distribución de insumos o en la propiciación de lo necesario para realizar un traslado se suman a los obstáculos impuestos por las desigualdades geográficas. 

La objeción de conciencia es indicada como un obstáculo en todos los informes. No hay una norma vinculante que estandarice y explicite este ejercicio para que las usuarias puedan evitar encontrarse con detractores en la consulta y ser violentadas. Algunas y algunos profesionales, en vez de explicitar su posición y derivarlas de buena fe, tratan de disuadirlas para que cambien de opinión y emiten juicios morales que no corresponden a su función. También se encuentran con ecografistas que las fuerzan a escuchar latidos o administrativas o enfermeras que no les dan turno o le niegan la atención. Hay instituciones en las que todo el personal objeta y las autoridades no garantizan el personal adecuado o las derivaciones. Esta situación es especialmente problemática en las zonas con escasos centros de atención en muchos kilómetros.

Las personas responsables de la gestión de la aplicación, ya sea a nivel nacional o provincial, pivotean entre las resistencias del personal de salud de los servicios y la exigencia de las activistas y en menor medida de las usuarias; se resisten a hacer públicos los servicios de salud que hacen los abortos, porque todos “deberían hacerlo”, lo que llevaría a la sobrecarga de estos servicios. Pero, de hecho, ya están sobrecargados porque hay una parte de las y los profesionales que sienten la potestad de elegir si hacer la práctica o no y, en definitiva, se sigue recurriendo a los espacios menos hostiles. Es decir que funcionarias y funcionarios, lejos de evitar la sobrecarga de los servicios amigables, lo que consiguen es que las usuarias también lleguen frustradas por haber sido rebotadas de lugar en lugar, y que las activistas reclamen mayor claridad. Si bien dos años de la implementación de una ley es un tiempo corto, se necesita marcar precedentes de qué actitudes son toleradas y cuáles no, porque las opiniones personales no son aceptables en cuestiones de ampliación de los derechos de las mujeres y colectivos LGTTBIQ+.

En las diferentes provincias, las activistas han emprendido acciones de acompañamiento para el acceso efectivo a la IVE/ILE, algunas respondiendo consultas individuales, otras a través de las redes sociales, y otras telefónicamente y hasta en persona. Han realizado acompañamiento y asesoramiento legal a médicas y mujeres judicializadas y monitoreos sociales para tener un panorama de la situación vigente y exigir mejoras en los servicios de salud. Desde su accionar, los obstáculos con los que se encuentran son la deficiencia de la información sobre los derechos y los cambios legales, pero también la falta de claridad de las acciones administrativas, la excesiva burocracia, el pedido de mayores requisitos que los establecidos, y la escasa incidencia de personal para fiscalizar a las obras sociales o al sistema privado.

Para el Estado, las consejerías y la información que las interesadas solicitan por fuera del sistema de salud no forman parte de los procesos de atención-cuidado, sin tener en cuenta que durante años fueron estas las que dieron información y acompañaron las situaciones de abortos. Cuando una persona tiene alguna consulta relativa a su salud recurre a quien considera que puede darle una respuesta. En el caso del aborto, las activistas se convirtieron cada vez más en quién recurrir para información y cuidados.

Lo principal que consultan las interesadas es adónde acudir para realizarse una IVE. Muchas señalan temor a ser maltratadas, juzgadas o criticadas. Con la ley, y un sistema de salud que se atribuye la atención de las IVE, se esperaría mayor difusión y poner a disposición todos los recursos necesarios para facilitar el acceso. Sin embargo, las mujeres suelen rebotar en los mostradores de información, y a las activistas no se les comparte la información actualizada de los efectores que garantizan la práctica sin maltratos o bien de quienes se niegan a realizarlo. 

Las demandas de información, además, tienen que ver con qué hacer o con cómo usar las pastillas si esto no ha quedado claro en la consejería. Para otras, el aborto no se produjo como esperaban. Otras quieren tener un apoyo para acudir durante el momento del aborto por si les da miedo o tienen alguna duda de algún síntoma. El protocolo señala qué hacer en los casos de síntomas de alarma, pero no establece canales claros de consultas una vez que se tienen las pastillas en la mano. Entonces cuando tienen dudas sobre el uso o sobre lo que están sintiendo, o cuando no ocurrió lo esperado, las mujeres acuden a las activistas, quienes no son reconocidas como parte integral del proceso por el sistema de salud.

## CONSIDERACIONES FINALES

En este artículo vimos cómo se entrelazan las barreras y desigualdades estructurales del sistema de salud con los obstáculos políticos que trae una práctica que ha sido clandestinizada por al menos un siglo. Las barreras simbólicas, económicas, geográficas, raciales y etarias se conjugan y le dan características particulares a la problemática de la implementación de la Ley IVE. En estos dos años ha habido voluntad política para promover su cumplimiento, no solo a nivel nacional, sino también en provincias y municipios, gracias al trabajo de acompañamiento y monitoreo de las activistas, a las redes de profesionales, al personal y a funcionarias y funcionarios de salud “amigables”. 

Los ejemplos de maltratos y obstaculizaciones aparecen mayormente en la intervención concreta. Con la legalidad de la práctica, al acudir a los servicios de salud se multiplican estas barreras: muchas son producto de que el personal de salud considera que puede obstaculizar el acceso sin consecuencias, y desde el Estado no interponen las sanciones necesarias para que dicho personal no traspase los límites de sus funciones. Al no exigirle al personal de la salud que se ajuste a la ley, los derechos de las y los profesionales tienen mayor peso que el derecho a la IVE de las mujeres y las personas con capacidad de gestar.

Si bien los informes muestran un incremento en la cantidad de servicios y profesionales que realizan las IVE/ILE en el sistema público, las activistas consideran que se deben tomar medidas más firmes para superar los obstáculos ideológicos y los maltratos de los detractores. Ningún servicio de salud debería tener la posibilidad de negarse a realizar la práctica porque prefiere no hacerlo. Esto ocurre cuando la implementación de políticas queda sometida a las voluntades personales, o cuando las creencias personales interfieren en las prácticas profesionales. Si bien las activistas en el sistema de salud crearon las condiciones para que en los servicios haya equipos capacitados para comenzar la implementación de la ley, esta debe ser considerada una parte integral de todo el proceso.
